# CAR T-cell design dependent remodeling of the brain tumor immune microenvironment identify macrophages as key players that inhibit or promote anti-tumor activity

**DOI:** 10.21203/rs.3.rs-2972427/v1

**Published:** 2023-06-06

**Authors:** Dalia Haydar, Jorge Ibañez-Vega, Jeremy Chase Crawford, Ching-Heng Chou, Cliff Guy, Michaela Meehl, Zhongzhen Yi, Deanna Langfitt, Peter Vogel, Christopher DeRenzo, Stephen Gottschalk, Martine F Roussel, Paul G. Thomas, Giedre Krenciute

**Affiliations:** 1 St. Jude Children’s Research Hospital, Department of Bone Marrow Transplantation and Cellular Therapy, Memphis, TN, USA; 2 Children’s National Hospital, Center for Cancer and Immunology Research, Washington, DC, USA; 3 St. Jude Children’s Research Hospital, Department of Immunology, Memphis, TN, USA; 4 St. Jude Children’s Research Hospital, Department of Tumor Cell Biology, Memphis, TN, USA; 5 University of Tennessee Health Science Center, Department of Microbiology Immunology Biochemistry, Memphis, TN, USA; 6 St. Jude Children’s Research Hospital, Department of Pathology, Memphis, TN, USA

**Keywords:** chimeric antigen receptor, CAR T-cell therapy, brain tumors, immunocompetent mouse models, scRNAseq, brain tumor immune microenvironment

## Abstract

Understanding interactions between adoptively transferred immune cells and the tumor immune microenvironment (TIME) is critical for developing successful T-cell based immunotherapies. Here we investigated the impact of the TIME and chimeric antigen receptor (CAR) design on anti-glioma activity of B7-H3-specific CAR T-cells. We show that five out of six B7-H3 CARs with varying transmembrane, co-stimulatory, and activation domains, exhibit robust functionality *in vitro*. However, in an immunocompetent glioma model, these CAR T-cells demonstrated significantly varied levels of anti-tumor activity. We used single-cell RNA sequencing to examine the brain TIME after CAR T-cell therapy. We show that the TIME composition was influenced by CAR T-cell treatment. We also found that successful anti-tumor responses were supported by the presence and activity of macrophages and endogenous T-cells. Together, our study demonstrates that efficacy of CAR T-cell therapy in high-grade glioma is dependent on CAR structural design and its capacity to modulate the TIME.

## INTRODUCTION

High-grade gliomas are the deadliest primary brain tumors, with a median survival of 15 months^1^. Current therapies including radiation, chemotherapy or surgery are unable to cure the disease and are often associated with serious adverse effects^2^. Immunotherapy using chimeric antigen receptor (CAR) T-cells offers a potentially safer and more effective alternative. CAR T-cells have shown promising results in preclinical brain tumor settings^3,4^; however, they have shown limited success in clinical trials^5–8^. The lack of success translating promising preclinical findings into viable clinical applications is in part due to the use of animal models that do not recapitulate human disease. Specifically, immunodeficient brain tumor models do not represent the complex immune niche. Thus, studies using immunocompetent mouse models are needed to address our limited knowledge of the brain tumor immune microenvironment (TIME) during CAR T-cell treatment.

The brain TIME is highly complex and heterogeneous^9,10^. Indeed, the TIME is dynamic and can change during tumor progression or in response to different therapeutic interventions^11,12^. However, mechanistic studies on how the TIME affects CAR T-cell functions remain limited. Understanding key factors within the TIME can lead to the identification of therapeutic targets that may enhance CAR T-cell effector function.

Lessons learned from previous studies show that careful selection of CAR functional domains is essential for effective CAR T-cell anti-tumor response^13–17^. Published data suggest faster and better activation of CD28-CARs, while 41BB-CARs are associated with improved overall persistence^18^. CD28-CARs signal through the PI3K-Akt pathway, whereas 41BB-CARs signal through the recruitment of TRAF proteins^19^. Additionally, calibrating CAR activation domains through inactivation of the first and third immunoreceptor tyrosine-based activation motifs (ITAMs) of CD3ζ results in decreased apoptosis and nonspecific activation of CAR T-cells^20^. Although the differential impact of CAR domains has been well established^21–23^, there are no studies evaluating the impact of different CAR functional domains on the composition and function of the TIME.

To address these gaps in knowledge, we generated a panel of murine B7-H3-specific CARs containing different transmembrane, costimulatory, and activation domains and compared their effector function *in vitro* and *in vivo*. We show that five out of six B7-H3-CARs with varying transmembrane, co-stimulatory, and activation domains, exhibit robust functionality *in vitro*. Interestingly, incorporating 41BBL^17,24^ signaling into our prototype 28.*m*ζ CAR T-cells^25^ significantly enhanced their expansion and persistence *in vitro*. However, these highly efficacious CAR T-cells showed poor anti-tumor responses *in vivo*. Single cell RNA sequencing (scRNAseq) analyses revealed distinctive changes in the TIME in response to different CAR constructs. We found immune cell clusters containing macrophages co-expressing both M1- and M2- genes (*Lyz2/Ccl8/Ccl6/Arg1/Mrc1/Thbs1)* to be associated with potent CAR T-cell responses. Indeed, global macrophage depletion using CSF1R inhibitors completely abrogated CAR T-cell therapeutic effects. Together, we show that successful tumor control by CAR T-cells is dependent on CAR structural design and requires functional T-cells and a balance of pro- and anti-inflammatory macrophages to prevent treatment failure and rapid CAR T-cell exhaustion.

## RESULTS

### Murine B7-H3 CAR T-cells have limited anti-glioma activity in immunocompetent models

We previously evaluated B7-H3 CAR T-cell therapy in an immunocompetent glioma model, where we demonstrated a significant survival advantage without signs of toxicity^25^. Following intra-tumoral injection of murine (m) B7-H3 CAR T-cells (28.*m*ζ) into C57BL/6 mice transplanted with GL261 tumor cells, only 60% of treated mice had complete responses (**Extended Fig. 1A-D**). The limited therapeutic responses were not associated with antigen downregulation, as evaluated by murine B7-H3 immunohistochemistry staining from non-responder mice (**Extended Fig. 1E-F**). Thus, we first sought to improve these CAR T-cells by optimizing CAR structure.

### Design and expression of mB7-H3 CARs with different domains

To investigate the role of CAR design in immunocompetent glioma models, we generated a panel of mB7-H3 CARs containing different transmembrane, costimulatory, and activation domains (**Fig. 1A and Supplemental Fig. 1A**). Initial studies^25^ included mB7-H3 CARs with CD28 transmembrane and costimulatory domains with mutated CD3ζ activation domain (**28.*m*ζ**). To first evaluate the effect of activation domains on anti-glioma activity of mB7-H3 CAR T-cells, we cloned a CD28-CAR with intact CD3ζ (**28.ζ**). As a control, we used an mB7-H3 CAR with a truncated endodomain (**Ctrl**). Additionally, we cloned two CARs with CD28 transmembrane domains and 41BB costimulatory domains, with a mutated or intact CD3ζ (**BB.*m*ζ** and **BB.ζ**). Similarly, we cloned two additional 41BB-CARs with a CD8 transmembrane domain (^**CD8tm**^**BB.*m*ζ** and ^**CD8tm**^**BB.ζ**). Lastly, we designed a CAR incorporating 41BB and CD28 costimulation through transgenic expression of 41BBL on the surface 28.*m*ζ CAR T-cells (**BBL-28.*m*ζ**).

CAR T-cells were generated by standard retroviral transduction as previously described^25^. We observed variable CAR expression on the surface of transduced murine T-cells (**Fig. 1B**) despite efficient expression of the 8 described constructs on GPE86 producer cell lines (**Supplemental Fig. 1B**). Moreover, the BB.ζ-CAR was consistently expressed at low levels on the T-cell surface and intracellularly (**Supplemental Fig. 1C**, Surface: 17.95 ± 2.46; Intracellular: 22.1± 1.41). Thus, this construct was excluded from additional testing. To ensure that differences in CAR expression did not confound our results, non-transduced (NT) T-cells were added to each product to achieve a final CAR-positive level of 40% (**Fig. 1C**, p<0.0001). All CAR T-cells were used immediately after CAR-positive level adjustment (**Supplemental Fig. 1D**).

### Functional characterization of mB7-H3-CARs with different domains

We next tested mB7-H3 CAR T-cell memory phenotype. We observed a comparable ratio of CD4 to CD8 T-cells among all constructs except for Ctrl-CAR, which had slightly higher CD4/CD8 ratio (**Fig. 1D**, p<0.05). Additionally, there were no significant differences in T-cell memory phenotypes for CAR-positive cells except for the BBL-28.*m*ζ transduced T-cells, which exhibited a slightly higher proportion of central memory phenotype (**Fig. 1E**, p<0.05).

To evaluate if different CAR domains can alter B7-H3 CAR T-cell cytotoxicity, we performed a 72-hour MTS assay. Cells were cocultured at different effector to target (E:T) ratios without exogenous cytokines. Cytolysis was then measured by colorimetric quantification of viable tumor cells. In contrast to controls (NT or Ctrl-CAR), all mB7-H3 CARs had efficient cytolytic activity against GL261 tumor cells at higher E:T ratios (**Fig. 2A**, p<0.0001). However, 41BB-based CARs with mutated CD3ζ (BB.*m*ζ and ^CD8tm^BB.*m*ζ) exhibited significantly reduced cytolytic activity at lower E:T ratios (**Fig. 2B**, NT and Ctrl vs BB.*m*ζ or ^CD8tm^BB.*m*ζ p<0.0001; BB.*m*ζ vs ^CD8tm^BB.*m*ζ p>0.05; BB.*m*ζ or ^CD8tm^BB.*m*ζ vs other constructs p<0.0001). Importantly, all CARs exhibited no cytotoxicity against B7-H3-negative target cells (GL261-KO; **Supplemental Fig. 1E**) confirming specificity.

### Surface expression of 41BBL provides expansion and persistence advantages

To evaluate if altering CAR domains can enhance the sequential killing and persistence of mB7-H3 CAR T-cells, we transduced T-cells with different mB7-H3 CAR constructs were cocultured with GL261 or GL261-KO at an E:T ratio of 2:1 without exogenous cytokines. If T-cells killed and expanded, they were restimulated with fresh tumor cells every 3 days. All mB7-H3 CAR T-cells killed and expanded for up to 2 stimulations against GL261 except for NT and Ctrl CAR T-cells (**Fig. 2C**). There were no significant differences in the maximum fold expansion among CARs with single costimulatory domains except for the ^CD8tm^BB.*m*ζ-CAR, which failed to expand or persist beyond 2 stimulations (**Fig. 2D**, ^CD8tm^BB.*m*ζ vs NT or Ctrl p>0.05; ^CD8tm^BB.*m*ζ vs other constructs p<0.05). Our prototype CAR (28.*m*ζ) had enhanced persistence averaging about 3-weeks in repeat-stimulation assays (6–7 sequential killings) vs 0.7–2 weeks for other constructs (2–5 sequential killings) (**Fig. 2E**, 28.*m*ζ vs ^CD8tm^BB.ζ p>0.05; 28.*m*ζ vs other constructs p<0.005). Remarkably, transgenic expression of 41BBL on the surface of 28.*m*ζ CAR T-cells provided a significant expansion and persistence advantage when compared to all other CAR T-cells (**Fig. 2D-E**, BBL-28.*m*ζ vs other constructs p<0.0001). Moreover, expansion was antigen-specific with no expansion or persistence upon stimulation against GL261-KO or media only (**Extended Fig. 2**).

### mB7-H3 CAR T-cells show sustained cytokine secretion upon multiple stimulations

To evaluate the effector functions of different mB7-H3 CARs, we measured cytokine secretion upon repeated-stimulation. Co-culture supernatants were collected at 24-hours after each stimulation, and the concentrations of Th1 (IFN-γ, IL-2, TNF-α, IL-1α, GM-CSF, IL-3) and Th2 (IL-4, IL-5, IL-6, IL-9, IL-10) cytokines and chemokines (LIX, MIP-1a, MIP-1b) were measured using a multiplex assay. Compared to NT or Ctrl CAR T-cells, all mB7-H3 CARs secreted significantly higher levels of cytokines after stimulation with GL261 except for ^CD8tm^BB.*m*ζ-CAR, which is consistent with expansion data (**Fig. 3A**, stim 1: NT and Ctrl vs ^CD8tm^BB.*m*ζ p>0.05, NT and Ctrl vs other constructs p<0.005). Importantly, BBL-28.*m*ζ CAR T-cells produced significantly higher levels of cytokines compared to T-cells transduced with other constructs (**Fig. 3A**, stim 1: BBL-28.*m*ζ vs other constructs p<0.005). With subsequent stimulations, BBL-28.*m*ζ CAR sustained higher levels of cytokine production (**Fig. 3B**, stim 4: BBL-28.*m*ζ vs other constructs p<0.0001). Specifically, BBL-28.*m*ζ CAR T-cells released higher levels of IFN-γ, IL-2, and GM-CSF at first and fourth stimulations with GL261 tumor cells (**Fig. 3C-E**, BBL-28.*m*ζ vs other constructs p<0.05).

### *In vitro* findings do not accurately predict *in vivo* performance

Next, we evaluated if optimizing CAR structure can enhance anti-glioma efficacy of mB7-H3 CAR T-cells *in vivo*. To avoid immunogenicity effects of luciferase and fluorescent proteins^26,27^, we used unmodified GL261 tumor cells. We used magnetic resonance imaging (MRI) to monitor tumor implantation and progression (**Fig. 4A-B**). On Day 0, GL261 cells were implanted into the brains of C57BL/6 mice, followed by intra-tumoral injection of 3 × 10^6^ CAR T-cells on Day 7. Treatment groups included Ctrl CAR and different mB7-H3 CARs depicted in **Fig. 1A**. *In vivo*, 28.*m*ζ CAR T-cells demonstrated the best overall control of tumor burden, which translated into enhanced survival (**Fig. 4C-D**, 28.*m*ζ vs ^CD8tm^BB.*m*ζ p>0.05; 28.*m*ζ vs all other constructs p<0.001). Interestingly, while ^CD8tm^BB.*m*ζ CAR T-cells did not perform well *in vitro*, they had comparable efficacy to the 28.*m*ζ CAR T-cells *in vivo* (**Fig. 4A-D**). Strikingly, BBL-28.*m*ζ CAR T-cells showed sub-optimal anti-glioma responses *in vivo* (**Fig. 4D**, BBL-28.*m*ζ vs 28.*m*ζ, ^CD8tm^BB.*m*ζ, or Ctrl p<0.05; BBL-28.*m*ζ vs all other constructs p>0.05). Collectively, our data suggests that presence of the brain TIME influences CAR T-cell *in vivo* performance (**Fig. 4E**).

### TIME composition during CAR T-cell treatment depends on CAR design

To investigate the TIME changes during CAR T-cell treatment, we performed scRNAseq analyses. Tumors were collected at 4-days post treatment, processed, sorted for CD45+ and CD45− fractions, and mixed at a ratio of 70% to 30%, respectively, for scRNAseq (**Fig. 5A**). We selected 4 CAR T-cell treatment groups that showed the best (28.*m*ζ, n=2), sub-optimal (BBL-28.*m*ζ, n=1 and ^CD8tm^BB.*m*ζ, n=2) and no (Ctrl, n=2) anti-tumor response (**Fig. 5A**, **Supplemental Fig. 2**). Bioinformatic analyses revealed five major cell types based on hallmark gene expression: myeloid (*Cd11b*; 12 clusters), lymphoid (*Cd33/Ncr1/B220/Cd19*; 5 clusters), tumor (*Olig2/Cd276/Col11a1*; 1 cluster), endothelial (*Enpp2*; 1 cluster) and fibroblast (*plp1/Ptgds*; 1 cluster) cells (**Fig. 5B**). The myeloid compartment constituted the largest proportion of all clusters, with no differences in frequency among treatment groups (**Fig. 5C**). We next identified 21 transcriptionally unique cell clusters, including 16 distinct immune cell populations (**Fig. 5D**). Cluster identities were confirmed using ImmGen murine cell ID database, corroborating those identities determined by canonical genes for each cell type (**Supplemental Fig. 3–4**). We observed no apparent differences in cluster frequencies per treatment group (**Fig. 5E**, **Extended Fig. 3A**). Thus, we next performed T-cell and macrophage sub-clustering analyses.

### T-cells infiltrating tumors that failed CAR T-cell therapy are predominantly exhausted

To analyze endogenous T-cell responses post CAR T-cell treatment, we further re-clustered the CD4 and CD8 lymphoid compartment (**Fig. 5D**, clusters 2, 8, and 16). T-cells diversified into 11 subclusters with unique transcriptional profiles (**Fig. 6A-B**, **Supplemental Fig. 5A**). First, tumors treated with Ctrl CAR had the largest proportion of naïve-like (C1: *Tcf7/Sell/Lef1*) and quiescent (C4: *S1pr1/Klf2/Slfn1*) T-cells (**Fig. 6C**). Second, BBL-28.*m*ζ and ^CD8tm^BB.ζ CAR-treated tumors were enriched in effector memory T-cells, C0 and C6 (**Fig. 6B**). Subclusters C0 and C6 expressed genes involved in immune effector processes like T-cell activation (*Cd28/C1qa/Lat*), cytokine signaling (*Zap70/Ifitm1/Ifngr1*), and cytotoxicity (*Gzma/Gzmb/Prf1*). However, these T-cells also expressed multiple inhibitory receptors (*Pdcd1*/*Ctla4*/*Lag3*/*Havcr2*), *Eomes*, and *Tox*. Third, tumors treated with ^CD8tm^BB.ζ-CAR had larger proportions of regulatory (C2: *Foxp3/Tnfrsf9*) and effector (C7: *Ifng/Ccl1/Ccl4*) CD4 clusters. These CD4 clusters upregulated genes associated with antitumor immunity and chemotaxis (*Lat/Prf1/Zap70/Ccl3*) while simultaneously expressing genes associated with inhibitory T-cell responses (*Lag3/Havcr2/Tox/Ctla4*) (**Fig.6C, Supplemental Fig. 5A**). Collectively, endogenous T-cell responses in BBL-28.*m*ζ and ^CD8tm^BB.ζ groups were associated with a hyper-activated T-cell phenotype along with a concurrent expression of inhibitory/exhaustion signatures (**Fig. 6D-E**). In contrast, we observed a balanced presence of immune-activating and immune-dampening genes in the 28.*m*ζ-CAR group which translated into protection against hyper-activation and exhaustion (**Fig. 6D-E**; **Supplemental Fig. 5B-C**).

Using computational approaches, we detected CAR antigen recognition domain (scFv) expression in macrophage, DC, and T-cell clusters (**Extended Fig. 3B**). Results suggest potential engulfing and uptake of CAR T-cells by antigen presenting cells. Focusing on T-cell subclusters, we found that CAR-positive cells within subclusters 3, 5, and 8 were mostly from tumors treated with 28.*m*ζ-CAR (**Fig. 6F**). Subcluster 3 differentially upregulated genes associated with proliferation and cell cycle progression (*Mki67/ Hist1h1d/Cenpa*) while subcluster 5 and 8 upregulated genes responsible for memory and cytolytic T-cell activity (C5: *Ly6c2*; C8: *Gzmc/Gzmf/Ccl1*). (**Fig. 6G-I**). Notably, ^CD8tm^BB.ζ-CAR was detected at low frequencies in subclusters C3 and C8 (**Fig. 6F**). Together, these data suggest that CAR T-cells post-treatment are highly proliferative, activated and cytolytic.

### Not all macrophage responses after CAR T-cell treatment are suppressive

Because macrophages are known to play an immunosuppressive role in the brain TIME^28^, we next sought to further characterize the macrophage and macrophage-related compartments in our dataset. Cell-cell communication analysis (CellChat^29^) using our scRNAseq dataset showed that macrophage/microglia (Mac/MG) clusters had the largest number and strength of interactions, suggesting their putative role in shaping the TIME post CAR T-cell treatment (**Extended Fig. 4**). We re-clustered macrophage (Mac) and macrophage/microglia (Mac/MG) populations (**Fig. 5D**, C0, 7, 10, and 14) resulting in 10 subclusters that included tissue-resident and monocyte-derived populations (**Fig. 7A-B**, **Supplemental Fig. 6A-B**). The latter were defined by *Itga4* expression versus microglia-related gene expression in tissue-resident subsets (*Tmem119*/*P2ry12*). While macrophage responses were heterogeneous, we observed that subclusters 2 and 7 were enriched in the 28.*m*ζ-CAR treatment group (**Fig. 7B**; C2: 21.77%, C7: 11.26%). Both subclusters expressed an array of anti- and pro- inflammatory genes simultaneously and were thus labeled as M1/M2 transitioning macrophages (**Fig. 7B-C**). Subcluster 2 upregulated genes associated with inflammation (*Lyz2/Apoc2/Ifngr2/Irf7*) and immune regulation (*Arg1/Ccl8/Spp1/Gpnmb*) (**Fig. 7C**). Similarly, subcluster 7 upregulated immunosuppressive (*Arg1/Mrc1/Chil3/Fn1*) and pro-inflammatory (*Cxcl2/Thbs1/Pf4/Il1b*) genes (**Fig. 7C**). Gene set enrichment analysis (GSEA) showed upregulation of hallmark pathways associated with inflammation (C2: complement, coagulation, reactive oxygen species; C7: inflammatory response, TNF signaling, glycolysis) along with downregulation of immunoregulatory pathways (C2 and C7: E2F targets, Myc targets, IFNalpha response) when compared to other subclusters (**Fig. 7D-E**). Moreover, comparing macrophage responses in 28.*m*ζ-CAR group showed significant upregulation of hallmark pathways related to immune response and downregulation of pathways related to immune suppression compared to other groups (**Fig. 7F**).

Lastly, we noticed interesting inverse patterns between 28.*m*ζ and Ctrl CAR treatments when looking at macrophage gene expression (**Fig. 7G**, **Supplemental Fig. 6C-D**). Specifically, genes associated with immunosuppression (*Apoe/Id2/Stat3*), M2-like phenotype (*Mrc1/Hif1a/Chil3*), and invasion (*Ctsb/Ctsd/Spp1)* were upregulated with Ctrl CAR compared to 28.*m*ζ-CAR treatment. Conversely, genes associated with M1-like responses (*H2-Ab1/H2-Eb1/H2-Aa*) were upregulated with 28.*m*ζ-CAR compared to Ctrl CAR treatment. Together, our data show distinctive macrophage responses post CAR T-cell treatment that correlate with successful anti-tumor responses only in CARs with specific costimulatory domains.

### Non-responding mice show dysfunctional and suppressive immune cells at endpoint

To further understand changes in the TIME that contribute to limited therapeutic efficacy of mB7-H3 CAR T-cells, we investigated the immune composition of tumors at endpoint. Tumor samples were collected from non-responding mice at the time of sacrifice and used for high-dimensional flow cytometry analyses (**Extended Fig. 5A**). Samples included normal brains (WT) and tumors treated with different mB7-H3 CARs (**Fig. 4D**). Importantly, 28.*m*ζ-CAR treated tumors were completely eradicated, thus this group was not part of endpoint analyses. We observed an overall increase in CD45+ immune cells in CAR-treated tumors compared to normal brains (**Extended Fig. 5B**). Specifically, there was an overall increase in macrophage, dendritic cell, B cell, NK cell, and T-cell infiltrates within the TIME (**Extended Fig. 5C**, **Supplemental Fig. 7**).

To identify distinct lymphoid populations, we stained tumor samples using a panel of comprehensive T-cell immunophenotyping markers and applied a multi-dimensional data-driven clustering algorithm to define unique populations (**Extended Fig. 5D-E**). Consistent with scRNAseq data from early timepoint, we found that mice treated with 41BB-CARs had higher proportions of T-cells at endpoint compared to mice treated with CD28-CARs. Specifically, data shows a larger proportion of central memory CD4+ T-cells in tumors treated with 41BB-CARs (**Extended Fig. 5D**, **Supplemental Fig. 8**). Additionally, larger percentages of CD8+ effector T-cells infiltrated tumors treated with BBL-28.*m*ζ CAR (**Extended Fig. 5E**, **Supplemental Fig. 8**). Yet, most T-cell subsets (including CAR T-cells) expressed multiple exhaustion markers indicating that persisting T-cells at endpoint are dysfunctional (**Extended Fig. 5D-E**, **Supplemental Fig. 8–9**).

To delineate specific phenotypes of infiltrating cells, we did multi-dimensional subclustering of ***myeloid populations (**Extended Fig. 5F**). We found that the majority of macrophages in non-responding mice were microglia-derived (MG-TAMs, *CD49d*^*lo*^)^30^. Similar to scRNAseq data from early timepoints, MG-TAMs in BBL-28.*m*ζ and ^CD8tm^BB.ζ groups intracellularly expressed M2-like suppressive markers (Arg1/Il10/Ahr) (**Supplemental Fig. 10A-C**). Additionally, M1/M2 transitioning macrophages concurrently expressing pro- and anti- inflammatory markers (Arg1/Il10/Ahr/Il17/Ifng/Tnfa/IL6) were also present at endpoint (**Supplemental Fig. 10A-C**). Thus, our FACS data from tumors at endpoint reiterate the potential role of endogenous myeloid and T-cell populations in dictating successful responses to CARs with distinct structural domains.

### Global macrophage depletion abrogates CAR T-cell anti-tumor efficacy

To investigate the contribution of macrophages to CAR T-cell efficacy, we pretreated mice with CSF1R inhibitor, BLZ945. This drug has been shown to effectively deplete spinal cord and brain macrophages as well as microglia in brain tumor models^31,32^. Importantly, we found that BLZ945 does not affect CAR T-cell functions *in vitro* (**Extended Fig. 6–7**). Daily treatment with 200mg/kg of BLZ945 in glioma-bearing mice effectively depleted all macrophages following 11-days of consecutive dosing (**Fig. 8A-B**, p=0.0005). Therefore, we evaluated whether 11-day pretreatment with BLZ945 would affect anti-glioma efficacy of 28.*m*ζ CAR T-cells. Interestingly, macrophage depletion had a negative impact on tumor control and survival in mice receiving BLZ945 in combination with mB-H3 CAR T-cells (**Fig. 8C-D, Extended Fig 8A**). Immunostaining of tumors from endpoint revealed that macrophages continue to be depleted in groups treated with BLZ945 while T-cell infiltration was not impacted (**Fig. 8E**, **Extended Fig 8B**). This translated into significantly lower protein expression of Iba1, F4/80, and Cd11c detected by immunohistochemistry (IHC) in BLZ945-treated tumors versus no significant differences in CD3 protein expression (**Fig. 8F**, **Extended Fig 8C**).

To confirm the detrimental effects of BLZ945 on CAR T-cell efficacy were not related to larger tumor volumes due to the delayed treatment timeline, we performed the same combination therapy but started BLZ945 treatment at 4-days prior to tumor implantation, and injected CAR T-cells intratumorally at 7-days post implantation (**Extended Fig 7D**). Similarly, global macrophage depletion continued to have a detrimental effect on CAR T-cell therapy in glioma-bearing mice (**Extended Fig 8E**). Together these data expand upon our TIME analyses to confirm that specific macrophage subsets are essential for CAR anti-tumor responses.

## DISCUSSION

The potential of T-cell immunotherapies to induce complete responses in brain tumors depends on their ability to elicit robust and sustained effector functions within the hostile TIME^33^. In addition, durable immune responses require differentiation of infused CAR T-cells into effector and memory phenotypes that allows them to persist in the highly suppressive microenvironment^34,35^. Here, we show that optimizing CAR design based on *in vitro* profiling is not sufficient to induce superior responses in the presence of the TIME. *In vitro* experiments demonstrate that 28.*m*ζ CAR T-cells have the best overall cytotoxicity and that incorporating 41BBL signaling in 28.*m*ζ CAR T-cells enhances their expansion and persistence. To our surprise, BBL-28.*m*ζ CAR T-cells did not have any superior survival advantage regardless of its phenomenal performance *in vitro*. Our scRNAseq analyses of tumors treated with CAR T-cells containing different functional domains revealed unique changes in the TIME composition. We found that two transcriptionally distinct macrophage clusters (subclusters 2 and 7) were enriched in tumors treated with the best functioning CAR (28.*m*ζ), suggesting that the presence of specific macrophage populations mediates successful CAR T-cell responses. Indeed, tumors depleted of macrophages and treated with 28.*m*ζ-CARs failed to respond to therapy.

It is well established that different CAR functional domains have strength and limitations when it comes to CAR T-cell effector functions. For example, CD28 domain provides faster and more profound activation of T-cell effector mechanisms due to enhanced changes in protein phosphorylation events compared to 41BB domain^36^. Moreover, CD3ζ can result in tonic CAR activation and early exhaustion^37^. Thus, calibrating CAR signaling by mutating one or two ITAMs can balance baseline CAR activation^38,39^. Finally, incorporating 41BB signaling into CD28-CARs using *in trans* design enhances therapeutic efficacy and persistence of CAR T-cells in leukemia and solid tumor models^40^. Our data reported here adds new knowledge to these existing findings. Specifically, we show that besides differential CAR T-cell signaling, the choice of CAR functional domains uniquely remodels the TIME and results in differential antitumor responses.

Macrophages heavily infiltrate gliomas and are associated with tumorigenesis and treatment resistance^41^. However, macrophage populations are highly heterogenous and perform specific functions ranging from enhancing tumor progression (enhanced tumor proliferation and invasion) to inducing immune evasion (restricting lymphoid infiltrates and promoting regulatory T-cell phenotypes). Moreover, macrophages are a major source of type I and II interferons which have direct antitumor effects and the potential to coordinate anti-inflammatory responses. Thus, macrophages are classified into classical (M1) and alternative (M2) phenotypes^42^. Although macrophage polarization is a dynamic state, M1-macrophages are considered pro-inflammatory and express markers associated with immune responses (Tnfa, Ifng, MhcII); while M2-macrophages are suppressive with characterized expression of immune regulatory markers (Arg1, Il-10, Cd206). In our study, we found 10 transcriptionally distinct macrophage subclusters present in tumors post CAR T-cell treatment and none of them fit perfectly in the classical M1- or M2- macrophage classifications reiterating the dynamic functions of macrophages within the TIME. As such, we find it essential to incorporate high-throughput analyses (such as scRNAseq and high-dimensional flow) into characterizing the brain TIME as it provides new insights into functional immune signatures. These findings may challenge traditional macrophage classification but will provide new insights into currently unknown macrophage functions within the brain TIME. It is important to replicate these studies for each unique tumor type to delineate macrophage interactions with adoptive immunotherapies in distinct tissues and etiologies.

In the past decade, an unprecedented number of genetic modifications have emerged significantly advancing T-cell based immunotherapies^43^. Most of them are aimed at improving T-cell signaling strength^44,45^, persistence and expansion^46,47^, or sensitivity to antigen density^48,49^. The standard approach for evaluating all new modifications is to first evaluate the improved functions *in vitro*, followed by *in vivo* testing in immunodeficient mouse models. Typically, *in vitro* findings translate into enhanced anti-tumor efficacy *in vivo*. However, only few studies verified new modifications in immunocompetent models^50–53^. Our study was exclusively performed in an immunocompetent brain tumor model using fully syngeneic CAR constructs. In this system, we found that *in vivo* results did not mirror our *in vitro* findings. Previous studies, including one from our group, showed that 41BBL does indeed improve CAR T-cell functions when tested in xenograft models^17,24^. These findings urge us to ask the following questions: Will all new CAR designs be evaluated in immunocompetent animal models for findings relevant to clinical applications? Does the brain microenvironment shut down highly potent CAR T-cells to protect it from CAR-induced inflammation? Are we over-engineering our CARs by making them more potent?

In conclusion, our results show that 28.*m*ζ-CAR have superior efficacy in immunocompetent glioma models. Analysis of the TIME revealed that presence of immune cell hubs consisting of macrophages and endogenous T-cells are associated with successful therapeutic responses to adoptive immunotherapies. Moreover, data from *in vivo* testing confirms that while helpful, *in vitro* results do not accurately predict how specific CAR domains function within the brain TIME. Finally, our study underscores the significance of investigating CAR T-cell functionality in models that have a functional immune system.

## MATERIAL AND METHODS

### Cell lines

The murine GL261 glioma cell line was purchased from DSMZ German Collection Laboratory, and the human embryonic kidney cell line 293T (or HEK 293T) and murine ecotropic retroviral packaging cell line GPE-86 were purchased from ATCC (Manassas, VA). The *B7-h3* knock-out GL261 (GL261-KO) cell line was generated using CRISPR-Cas9 technology as described in^25^. GL261 murine tumor cells were maintained in complete RPMI media (GE Healthcare Life Sciences, Chicago, IL) with 10% fetal bovine serum (FBS), 1% glutamax, 1% penicillin/streptomycin, 1% sodium pyruvate, 1% MEM Non-Essential Amino Acids, and 0.1% of 2-mercaptoethanol. GPE86 and 293T cells were maintained in DMEM media (GE Healthcare Life Sciences, Chicago, IL) with 10% FBS, 1% glutamax, and 1% penicillin/streptomycin. Additionally, murine T-cells were maintained in RPMI media with 10% FBS, 1% glutamax, 1% penicillin/streptomycin, 1% MEM non-essential amino acids, and 0.1% of 2-mercaptoethanol. All cell cultures were screened for mycoplasma by using MycoAlert Mycoplasma Detection Kit (Lonza, Walkersville, MD) every 3–4 months. Cell lines were kept for 2–3 months (or up to 12 passages) in culture before they were replaced by a fresh vial of cells. All cell lines were authenticated using IDEXX Bioanalytics (Westbrook, ME).

### Animal models

All animal experiments followed a protocol approved by the St. Jude Children’s Research Hospital Institutional Animal Care and Use Committee. Mice were euthanized when they met physical euthanasia criteria (significant weight loss, signs of distress), or when recommended by St. Jude veterinary staff. Albino C57BL/6 mice (B6(Cg)-*Tyr*^*c−2J*^/J, 000058, The Jackson Laboratory) were purchased at 6–8 weeks of age. All animals used for tumor implantation were 10–12-weeks old males or females. For intracranial implantation, mice were anesthetized and placed in a stereotactic rodent surgery platform. A 1-mm burr hole was drilled into the skull at 1mm anterior and 2mm to the right of the bregma. A total of 1×10^5^ GL261 cells in 2μL of media were injected at 3-mm deep as described in^25^. Wound clips were then used to close the surgical site. On Day 7 post tumor implantation, animals were treated with 3×10^6^ effector cells adjusted to 40% CAR expression and suspended in 2μL of media via intra-cranial injection using the same burr hole coordinates. Each experiment involved 5 mice per group and biological replicates were done using CAR T-cells generated from different donors.

For experiments with BLZ945 combination, oral treatment was started on day 5 post tumor implantation or at 4-days prior. Briefly, BLZ945 (TargetMol, Wellesley Hills, MA) was resuspended in 20% Captisol (Captisol, San Diego, CA) at 60mg/mL. Mice were dosed at 200mg/kg in 100uL via oral gavage. Control treated mice received 20% Captisol. Treatment continued daily for 3–4 weeks, and fresh drug formulation was prepared every other day for the duration of treatment.

### Generation of murine CAR vectors

To generate mB7-H3 CAR T-cells, we designed retroviral vectors encoding second-generation CARs with an antigen recognition domain derived from the mB7-H3-specific monoclonal antibody m276^54^. We cloned 6 constructs: (1) ^CD28tm^CD28.ζ; (2) ^CD28tm^41BB.ζ; (3) ^CD8tm^41BB.ζ; and (4–6) same as (1–3) with two additional mutations in the first and third ITAMs of the CD3ζ signaling domain as described in^20^. As a control, we cloned a nonfunctional mB7-H3 CAR without a signaling domain. Finally, to generate CD28-based CARs expressing 4–1BBL on the T-cell surface, we cloned a vector encoding murine 41BBL, a 2A sequence, and the CD28-CAR with mutated CD3ζ domain. All final CAR vectors were verified by sequencing.

### Generation of retroviral particles for murine T-cell transduction

Retroviral particles for murine T-cell transduction were generated from GPE-86 producer cell lines expressing the 8 different CARs as described in^25^. Briefly, viral supernatant was first generated from transient transfection of 293T cells with the CAR-encoding vector, Peg-Pam plasmid encoding MoMLV gag-pol, and a plasmid encoding the VSVG envelope. The VSVG-pseudotyped viral particles and polybrene (8mg/mL, Millipore Sigma, Burlington, MA) were then used to transduce the GPE-86 producer cell line. The transduced GPE-86 cells were then stained for CAR expression (AF-647 anti-human IgG, F(ab′)_2_ fragment-specific) and FACS sorted (BD FACSAria III) to isolate the top 50% CAR positive cells. Fresh supernatant from the producer cell lines was generated prior to each CAR T-cell transduction experiment by maintaining the sorted producer cell lines in culture in IMDM media (GE Healthcare Life Sciences, Chicago, IL) supplemented with 10% FBS, 1% glutamax, and 1% penicillin/streptomycin.

### Generation of murine CAR T-cells

mB7-H3 CAR T-cells were generated using standard retroviral transduction of murine T-cells as described in^25^. Briefly, CD3+ T-cells were enriched from murine splenocytes using the Pan T-Cell Isolation Kit (Miltenyi Biotec, Bergisch Gladbach, Germany). Spleens were collected from 6–8 weeks old C57BL/6 mice (C57BL/6J, 000664, The Jackson Laboratory, Bar Harbor, ME) and passed through a 70-μm cell strainer using the back of a syringe plunger. Red blood cells were then lysed using ACK lysis buffer (A10492–01, Life Technologies, Carlsbad, CA), washed and pelleted. CD3+ murine T-cells were then collected using the Pan T-Cell Isolation Kit II (130–095-130, Miltenyi Biotec) as per the manufacturer’s protocol. Isolated T-cells were washed and activated using plate-bound CD3/CD28 antibodies (553057 and 553294 respectively, BD) in complete RPMI media supplemented with 50U/L of human IL-2 (Peprotech, Rocky Hill, NJ).

At 48-hours post-activation, T-cells were transduced with pseudotyped retroviral particles on RetroNectin-coated plates (Clontech, Mountain View, CA) in complete RPMI media supplemented with 50 U/mL of IL-2. NT T-cells were prepared by adding activated T-cells on RetroNectin-coated wells without retroviral particles. CAR T-cells were then harvested 48-hours later and expanded in the presence of IL-2 (50U/mL), IL-7 (10ng/mL), and IL-15 (5ng/mL) (Peprotech, Rocky Hill, NJ) until Day 7 post-transduction. CAR detection was performed at Days 3–5 using flow cytometry. *In vitro* and *in vivo* experiments were performed at or before 9 days post-transduction.

### Functional analysis of murine CAR T-cells *in vitro*

#### Repeat stimulation assay

For serial co-culture assays, 5×10^5^ tumor cells (GL261 or GL261-KO) were plated in a 24-well tissue culture plate and allowed to adhere for 4–6 hours at 37°C. T-cell cultures were then prepared by washing cells twice with cytokine-free media and adjusting CAR expression to 40% by adding NT T-cells to CAR products with original CAR expression above 40%. A total of 1×10^6^ T-cells were then added to the wells containing tumor cells [2:1 E:T ratio]. At 3 days post-stimulation, T-cells were lifted by gentle pipetting and the number of T-cells was determined using a hemocytometer. For repeated stimulations, fresh tumor cells were plated and maintained in culture with T-cells at the same E:T ratio. Stimulations were repeated every 3 days until T-cells stopped killing and/or expanding.

For experiments including BLZ945 treatment, drug stock (953769-46-5, AbMole, Houston, TX) of 10mM in DMSO was prepared and checked for purity and identity using NMR and liquid chromatography–mass spectrometry. Working concentrations were then made in complete media and added directly after coculture setup for a final desired concentration of 67, 670, and 6700nM. Control wells were treated with equivalent concentrations of DMSO-containing media.

#### Cytotoxicity assay

Colorimetric MTS reagent [3-(4,5-dimethylthiazol-2-yl)-5-(3-carboxymethoxyphenyl)-2-(4-sulfophenyl)-2H-tetrazolium] (Promega, Madison, WI) was used to quantify the cytotoxicity of CAR T-cells, as described previously in^25^. Briefly, GL261 and GL261-KO tumor cells were added to a 96-well tissue culture-treated plate and allowed to adhere for 4–6 hours. T-cell cultures were washed to remove exogenous cytokines, adjusted to 40% CAR expression, and added to the tumor cells at different E:T ratios (4:1, 2:1, 1:1, 0.5:1, and 0.25:1). Co-cultures were then allowed to incubate for 72-hours at 37°C. Wells containing media or tumor cells only were used as controls to determine the absorbance corresponding to background and no T-cell killing, respectively. All conditions were done in triplicates. Experiments with BLZ945 treatment were done using media prepared with working drug concentrations described above. At 72-hours, T-cells were gently lifted and removed by pipetting up and down. MTS reagent was then diluted in complete media and added to each well, incubated at 37°C for 2 h and absorbance was then measured at 492 nm by using an Infinite® 200 Pro MPlex plate reader (Tecan, Männedorf, Switzerland). The percentage of live tumor cells was calculated using the following formula: [(Absorbance of sample-Absorbance of media only)/ (Absorbance of tumor only - Absorbance of media only)] ×100.

#### Cytokine production

At each stimulation of the repeated-stimulation assay, supernatant was collected 24 hours after plating T-cells with tumor cells and snap-frozen at −80°C for later analysis of cytokine production. Cytokine production was assessed by a 32-plex murine cytokine quantification kit (MCYTMAG-70K-PX32, Millipore Sigma) with analysis performed using a Luminex FlexMap 3D instrument and software (Luminex Corporation, Austin, TX).

### Flow Cytometry

All basic flow cytometry data were acquired with a BD FACSCanto II instrument and analyzed using FlowJo software (FlowJo, Ashland, OR). Samples were washed with and stained in DPBS (Lonza, Basel, Switzerland) with 1% HyClone FBS (Fisher Scientific, Hampton, NH). For all samples, matched isotypes or known negative (NT T-cells) served as gating controls. Invitrogen LIVE/DEAD^™^ Fixable Aqua Dead Cell Stain Kit (L34957, Fisher Scientific, Hampton, NH) was used as a viability dye.

For comprehensive TIME analysis, flow cytometry data was collected using a BD FACSymphony A5 cytometer (BD, San Jose) equipped with 355, 400, 440, 488, 561, and 640 nm lasers and 30 fluorescent detectors with long-pass and band-pass filters appropriate to the experimental fluorochromes.

#### T-cells and tumor cell lines

For analysis of B7-H3 expression on tumor lines, cells were stained with CD276-AF647 (562862, clone MIH32, BD, Franklin Lakes, NJ). For T-cell phenotyping, cells were stained with CD4-APC-Cy7 (552051, BD), CD8-FITC (553030, BD), CD62L-PerCpCy5.5 (560513, BD), and CD44-PE (553134, BD). The B7-H3-CAR was detected with AF647-anti-human IgG, F(ab′)_2_ fragment-specific antibody (109-606-006, Jackson ImmunoResearch, West Grove, PA).

#### TIME analysis

Brain tumor samples were harvested after mice were humanely killed via CO2 inhalation followed by decapitation. Briefly, brains were excised from the skull and the upper right quadrants of each brain including the tumor and surrounding normal tissue were collected in 2 mL of RPMI with 5% FBS. Brain tumor samples were then minced with scissors and incubated with 1mg/mL of collagenase IV (STEMCELL, Cambridge, MA) and 50U/mL of DNase I (Fisher Scientific, Hampton, NH) for 1 hour at 37°C. After enzymatic digestion, samples were then pushed through a 70μm cell strainer with a syringe plunger to create a single cell suspension. The samples were then centrifuged, and the pellet was suspended in 10mL of RPMI with 5% FBS. Cells were counted prior to being aliquoted into FACS tubes for processing for flow cytometry analysis.

Samples were first labeled with Live/Dead Fixable Blue dead cell stain (Fisher Scientific, Waltman, MA) according to the manufacturer’s protocol, then blocked against non-specific antibody binding through incubation with excess purified IgG (Fc Block; Biolegend, San Diego, CA). After washing, separate aliquots of cells were resuspended in either the lymphoid (19-antibody) or myeloid (21-antibody) optimized staining cocktails as outlined in Supplemental Table 1. Cells were typically incubated for 20 minutes on ice before washing and resuspending in a minimal volume of staining buffer for flow cytometric analysis.

For cytokine expression profiling of tumor infiltrates, cell samples were first stimulated in culture with optimized concentrations of PMA and ionomycin in the presence of monensin for 4 hours (Cell Activation Cocktail with Brefeldin A; Biolegend, San Diego, CA). Stimulated cell suspensions were then washed in PBS, labeled with Live/Dead Blue, and blocked with excess IgG, as described for surface immunophenotyping above. These samples were then incubated with the 11-antibody extracellular staining cocktail detailed in ST1 for cytokine expression profiling, and washed, before fixation and permeabilization with eBioscience Foxp3/transcription factor staining set (Fisher Scientific, Waltman, MA) according to the manufacturers protocol. Fixed and permeabilized cells were then incubated with the 9-antibody cocktail for intracellular staining (ST1), washed, and resuspended in a minimal volume of staining buffer for flow cytometric analysis.

Traditional layered bivariant analyses were conducted on the data collected using FACS Diva software for canonical cell surface markers as shown in Supplemental Table 2 (BD Biosciences, San Jose); however, most detailed information about marker expression patterns in the tumor infiltrates was obtained after T-Distibuted Stochastic Neighbor Embedding (t-SNE) algorithms were applied with the aid of FlowJo software (BD Biosciences, San Jose).

### Single-cell RNA sequencing

Brain tumors were collected and processed for FACS sorting using enzymatic digestion as described above. Single cells were then counted and stained in PBS with 5% FBS. Briefly, vials of TotalSeq-B antibodies were spun down and resuspended in 50uL of staining buffer. Cells were first stained with Fc Block (Biolegend, San Diego, CA) for 10 minutes at 4 °C. TotalSeq-B antibodies and flow cytometry antibodies (CD45-PE, Ter119-APC) were then added, and cells were incubated for 30-minutes at 4°C followed by washing with staining buffer. DAPI was used for dead cell staining prior to sorting and two populations were sorted (CD45- TER119- and CD45+ TER119-) into RPMI media with 10% FBS. Viable CD45+ and CD45− populations were mixed at 70:30 ratio and processed on the Chromium system (10X Genomics, 3’ v3) with a target of 10,00 cells per reaction. Gene expression and cell surface libraries were prepared according to the 10x manufacturer protocols. Sequencing was then performed on the Illumina NovaSeq 6000 platform.

### Bioinformatic analyses

#### Raw data processing and data visualization

Raw sequencing data were processed using CellRanger (10X Genomics, v6.1.2) with the corresponding mm10–2020-A reference. Individual reactions were then aggregated for read-normalization resulting in a mean of 43,828 post-normalized reads per cell. Downstream analyses were conducted with Seurat (v4.2.0)^55^ in R, retaining only genes found in at least 50 of the 42,105 post-aggregation cells. Dead and dying cells were excluded by removing those with at least 10% of gene expression owed to mitochondrial genes, and cells with fewer than 300 genes detected were likewise excluded. To remove putative multiplets, we excluded cells from each reaction that exhibited detected genes or RNA molecules at or above the 98% quantile for that respective reaction. Filtered expression data were subsequently normalized with default parameters. For clustering and dimensionality reduction, variable features were identified using the “vst” method, data were scaled without regression of other variables, and the top 1,000–3,000 variable genes were utilized for Principal Component Analysis (PCA) depending on the subset of data being analyzed. For macrophages and whole sample cluster analyses 1000 genes were used, and 3000 genes for T-cell clusters. UMAP dimensionality reduction and clustering utilized the first 7–30 principal components, depending upon the subset of the data under analysis.

To identify the cell identity of each cluster, we used the R package SingleR^56,57^, coupled to cellDex^56^ using the Immunological Genome Project^58^. Then, all the cell identities were corroborated based on expression of the reported/canonical markers.

Differentially upregulated genes (DEG) were performed using Seurat package, and for gene set enrichment analysis (GSEA), we utilized the Molecular Signature Database (MSigDB) by using the MSigDBr package in R^59^. All terms with a P<0.05 were considered significant and ranked by the number of genes identified in the group.

To infer cell-cell interaction in the scRNAseq data, we used the R package CellChat^29^. Briefly, we used the mouse ligand-receptor database, and inferred the cell-cell communication at the signal pathway level, to further aggregate the cell-cell communication. The most influential cell cluster was considered as the cluster that has the highest values in interaction as a sender/source.

To detect evidence of CAR transcripts, a *post hoc* analysis was performed by creating a custom reference comprised of mm10–2020-A and 729 bases of sequence from the CAR vector. Raw sequencing data were reprocessed with CellRanger v7.1.0 using this custom reference, and the resulting CAR UMI counts were extracted from the raw feature barcode matrices without read normalization and integrated into the Seurat objects used for previous analyses.

### Magnetic resonance imaging

Mice were imaged by the St. Jude Center for In Vivo Imaging and Therapeutics (CIVIT). MRI was performed on a Bruker Biospec 94/30 MRI system (Bruker Biospin MRI GmbH, Ettlingen, Germany). Prior to scanning, mice were anesthetized in a chamber (3% Isoflurane in oxygen delivered at 0.5 L/min) and maintained using nose-cone delivery (1–2% Isoflurane in oxygen delivered at 0.5 L/min). Animals were provided thermal support using a heated bed with warm water circulation and a physiological monitoring system to monitor breath rate. MRI was acquired with a mouse brain surface receive coil positioned over the mouse head and placed inside an 86mm transmit/receive coil. After the localizer, T2-weighted Rapid Acquisition with Refocused Echoes (RARE) sequences were performed in the coronal (TR/TE = 2000/20.4 ms, matrix size = 256 × 256, field of view = 20mm × 20mm, slice thickness = 0.5mm, number of slices = 16) and axial (TR/TE = 2500/23 ms, matrix size = 256 × 256, field of view = 20mm × 20mm, slice thickness = 0.5mm, number of slices = 32) orientations.

### IHC and H-score determination

Brains were perfused and fixed in 10% neutral buffered formalin, embedded in paraffin, sectioned at 5mm, mounted on positively charged glass slides (Superfrost Plus, Thermo Fisher, Waltham, MA), and then dried at 60°C for 20 min before dewaxing and staining with hematoxylin and eosin (H&E) using standard methods. For immunohistochemical detection of CD276 (B7-H3), CD3, and IBA1, tissue sections underwent antigen retrieval in a prediluted Cell Conditioning Solution (CC1) (Ventana Medical Systems, Oro Valley, AZ) for 30 min. Antigen retrieval for F4/80 and CD11c used Epitope Retrieval solution 1 (ER1) for 20 min and Epitope Retrieval solution 2 (ER2) for 30 min at 100°C respectively, both on a Bond Max immunostainer (Leica Biosystems, Buffalo Grove, IL). The primary antibody used to detect CD276 (B7-H3) was diluted 1:200 (AF1027, R&D Systems, Minneapolis, MN), with the OmniMap anti-rabbit HRP kit (Ventana Medical Systems, Oro Valley, AZ). The two different antibodies used to detect macrophages included anti-IBA1 (1:300 dilution, CP290A; Biocare Medical, Pacheco, CA) and rabbit anti-F4/80 (1:750, 70076; Cell Signaling, Danvers, MA). The other antibodies included anti-CD3 to detect T-cells (1:500 dilution, sc-1127; Santa Cruz Biotechnology, Dallas, TX) and CD11c (1:200 dilution, 97585, Cell Signaling, Danvers, MA).

H-scores were determined by using the HALO automated image analysis program (v3. 2.1851. 3, Indica Labs, Albuquerque, NM) on whole slide digital images to measure the intensity and extent of CD276 (B7-H3)-specific staining in tumors. Briefly, the HALO cytonuclear image analysis algorithm was first optimized and then run using tissue classifiers and annotations, to capture the percentage of cancer cells showing strong (3+), moderate (2+), weak (1+) or negative staining to calculate CD276 (B7-H3) H-scores for each sample. All sections were examined by a pathologist blinded to the experimental group assignments.

### Data Availability

Raw sequencing data for single-cell expression experiments have been deposited in the SRA under BioProject Accession PRJNA955817 and will be released upon publication. Reviewer can access the accession metadata at: https://dataview.ncbi.nlm.nih.gov/object/PRJNA955817?reviewer=1804u1umjlugif75v1h3bera0s [dataview.ncbi.nlm.nih.gov].

### Statistical analyses

All experiments were performed at least in duplicates. For comparisons between 2 groups, a 2-tailed *t*-test was used. For comparisons of 3 or more groups with a single independent variable, statistical significance was determined by one-way ANOVA with Tukey’s multiple comparisons test. For comparison of three or more groups with 2 or more independent variables, statistical significance was determined by multiple t-tests or 2-way ANOVA with Sidak’s or Tukey’s multiple comparisons test. Survival curves were plotted using the Kaplan-Meier method. Statistical significance between survival curves was determined using the log-rank (Mantel-Cox) test. Bioluminescence imaging data were analyzed using either ANOVA or t-test. P-values were calculated using Prism (GraphPad Software, San Diego, CA). * p<0.05; ** p<0.01; *** p<0.001; **** p<0.0001; ns, non-significant.

## Figures and Tables

**Figure 1.** Generation and functional characterization of syngeneic B7-H3 CAR T-cells with different CAR structures. **(A)** Scheme of murine (m) B7-H3-CAR constructs. **(B)** Summary plot of %F(ab′)2-positive T-cells at 3–5 days post transduction. **(C)** Summary plot of %F(ab′)2-positive T-cells titrated to 40% CAR expression with NT T-cells (n = 7, mean ± SD, 1-way ANOVA with Tukey’s test for multiple comparisons). **(D)** Summary plot of CD4 and CD8 composition of mB7-H3-CAR products at day 5 post-transduction (n = 4, mean ± SD, 2-way ANOVA with Tukey’s test for multiple comparisons). **(E)** Summary plot of memory phenotypes (Effector memory (EM): CD44+/CD62L−, central memory (CM): CD44+/CD62L+, naïve (N): CD44−/CD62L) of mB7-H3-CAR products as determined CD44 and CD62L expression (n = 4, mean ± SD, 2-way ANOVA with Tukey’s test for multiple comparisons).

**Figure 2.** CD28-based mB7-H3 CAR T-cells with mutated activation domains outperform other constructs in *in vitro* functional assays. **(A)** MTS cytotoxicity assay against GL261 tumor cells at an effector to target (E:T) ratio of 4:1 and **(B)** 0.25:1 (n = 7, mean ± SD, 2-way ANOVA with Tukey’s test for multiple comparisons). (C-E) T-cells expressing different mB7-H3 CAR constructs were cocultured with GL261 tumor cells at a 2:1 ratio with restimulation every 3-days against fresh tumor cells until they no longer killed and/or expanded. **(C)** Fold expansion of different T-cell donors upon successive stimulations (x-axis: each stimulation is a 3-day co-culture with fresh GL261 tumor cells, n = 7). **(D)** Summary of the maximum fold expansion of mB7-H3 CAR T-cells from individual donors upon repeat stimulation with GL261 tumor cells (n = 7, minimum to maximum range, 1-way ANOVA with Tukey’s test for multiple comparisons). **(E)** Maximum number of times CAR T-cells were able to kill Gl261 tumor cells (n = 7, minimum to maximum range, 1-way ANOVA with Tukey’s test for multiple comparisons).

**Figure 3.** Surface expression of 4–1BBL on CD28-based mB7-H3-CAR T-cells enhances effector cytokines release in repeat stimulation assay. Culture supernatants were collected at 24-hour post repeated-stimulation with GL261 tumor cells at 2:1 ratio and analyzed using Multiplex assay. **(A)** Summary plots of cytokines and chemokines produced by CAR T-cells post first stimulation and **(B)** fourth stimulation against GL261 tumor cells (n = 4, mean ± SEM, 2-way ANOVA with Tukey’s test for multiple comparisons). **(C-E)** CAR T-cell production of IFNγ, IL-2, and GM-CSF at 24 hours’ post-stimulations one and four (n = 4, mean ± SEM, 2-way ANOVA with Tukey’s test for multiple comparisons).

**Figure 4.** CAR structural design significantly impacts anti-glioma efficacy of mB7-H3 CAR T-cells in the GL261 immunocompetent model. Albino C57BL/6 mice were transplanted with 1×10^5^ GL261 cells orthotopically, followed 7-days later by intra-tumoral injection of 3×10^6^ mB7-H3-CAR T-cells transduced with different constructs and adjusted to 40% CAR expression. **(A)** Axial brain MRI images from 3 representative mice per treatment group at days 16 and 29 post-tumor implantation. **(B)** Summary plots for change in tumor volumes as measured by MRI at days 16 and 29 post-tumor implantation with lines connecting individual mice (n denotes the number of mice surviving at the day of imaging in each group). **(C)** Bar graph showing percentage of survival and deceased mice within each treatment group at days 16, 29, and 45 post-tumor implantation **(D)** Kaplan-Meier survival curve (n = 11, log-rank Mantel-Cox test with Bonferroni’s correction for multiple comparisons, *P<0.05; ***P<0.001). Experiments were repeated twice with CAR T-cells generated from 2 different T-cell donors. **(E)** Summary table for performance of different mB7-H3 CAR designs from *in vitro* and *in vivo* data ((−) means no response, increasing number of (+) signs mean better response).

**Figure 5.** Tumor immune microenvironment heterogeneity post CAR T-cell treatment. **(A)** Experimental scheme. Albino C57BL/6 mice were transplanted with 1 × 10^5^ GL261 cells orthotopically, followed 25-days later by intra-tumoral injection of 3 × 10^6^ mB7-H3-CAR T-cells (28.*m*ζ, BBL-28.*m*ζ, ^CD8tm^BB.ζ, or Ctrl). Tumors were collected at 4-days post treatment and processed for single-cell RNA sequencing. **(B)** UMAP with major cell subsets in all tumor samples. **(C)** Bar graph showing the percentage of each major cell type per treatment group. **(D)** UMAP dimensionality reduction of single cell data from all tumors clustered into 21 Seurat clusters annotated by number. **(E)** UMAP visualization of the 21 Seurat clusters by treatment group. Mac – macrophages, Mono – monocytes, MG – microglia, DC – dendritic cells.

**Figure 6.** Diversity of endogenous T-cell responses within the glioma TIME post CAR T-cell treatment. Seurat clusters 2, 8, and 16 were reclustered to further analyze lymphoid responses post CAR T-cell treatment. **(A)** UMAP plots of the T-cell subclusters visualized by treatment group. **(B)** Summary plot of T-cell subcluster distribution per treatment. **(C)** Dot plot depicting T-cell lineage and differentiation markers, T-cell immune inhibitory genes, T-cell cytolysis and immune activation genes, and chemotaxis genes per T-cell subcluster. Dot size represents the percentage of cells expressing each gene and dot color represents mean expression level with a gradient of lowest expression in blue to highest expression in red. **(D-E)** Dot plots depicting differentially expressed genes associated with T-cell immune activation and inhibition/exhaustion per treatment group. **(F)** Summary plot showing expression of CAR molecules per T-cell subclusters. **(G-I)** Volcano plots showing differentially –up and –down regulated genes in T-cell subclusters C3 in **(G)**, C5 in **(H)**, and C8 in **(I)** as compared to all other T-cell subclusters. Tex – Exhausted T-cells, Treg – Regulatory T-cells, Teff– Effector memory T-cells, Tquin – Quinescent T-cells, Trm – Tissue resident memory T-cells, Prol. – Proliferating.

**Figure 7.** Effective responses with 28.*m*ζ CAR T-cells are associated with balanced pro- and anti- inflammatory myeloid cell responses. Seurat clusters 0, 7, 10, 14 were reclustered into 10 macrophage/microglia subclusters to further define the diversity of myeloid responses post CAR T-cell treatment. **(A)** UMAP plots of the macrophage/microglia subclusters visualized by treatment group. **(B)** Summary plot of Mac/MG subcluster distribution per treatment. **(C)** Dot plot depicting expression of myeloid lineage markers, genes associated with pro-tumorigenic responses, anti-tumorigenic, and differentiation genes. Dot size represents the percentage of cells expressing each gene and dot color represents mean expression level with a gradient of lowest expression in blue to highest expression in red. **(D)** Enrichment plots of top six hallmark pathways involved in pro- and anti- inflammatory macrophage functions from GSEA Hallmark analysis comparing macrophage subcluster 2 versus other macrophage subclusters. Ranked genes depicted on the x-axis with a black line with most enriched on the left to least enriched on the right. Normalized enrichment score (NES) depicted as well as adjusted p-value (p. adjusted). **(E)** Enrichment plots for macrophage hallmark pathways enriched in subcluster 7 as compared to other macrophage subclusters. **(F)** Volcano plot showing differential gene expression profiles in macrophage subclusters associated with 28.*m*ζ-CAR treatment compared to all other groups along with dot plot for top 10 differentially up- or down- regulated hallmark pathways in macrophage subclusters associated with 28.*m*ζ-CAR treatment compared to other CAR groups. **(G)** Dot plot depicting differentially expressed genes associated with immunosuppression, invasion, recruitment, M1-like and M2-like macrophage responses per treatment group.

**Figure 8.** Global macrophage/microglia depletion abrogates effective CAR T-cell responses. GL261 glioma-bearing mice were treated with BLZ945 at 200mg/Kg starting 5-days post tumor implantation. **(A)** Experimental scheme of BLZ945 macrophage depletion kinetics experiment. Daily drug dosing via oral gavage was for 2 weeks and tumors were harvested for FACS analysis at days 9, 16, and 20 post tumor implantation. **(B)** Summary plot showing frequency of TAMs infiltrating tumors as percentage of live CD45+ immune cells. **(C)** Experimental scheme for combination study. Glioma-bearing mice were treated with BLZ945 at 200mg/Kg starting 5-days post tumor implantation and continued daily for 3 weeks. B7-H3 CAR T-cells with 28.*m*ζ domains were then injected intratumorally at day 16. **(D)** Kaplan-Meier survival curve (n= 11, log-rank Mantel-Cox test with Bonferroni’s correction for multiple comparisons, ***P<0.001). **(E)** Representative images from immunostaining for T-cell and macrophage markers from tumors at endpoint showing CD3 and Iba1 staining in brain samples from each treatment group at 40x magnification (scale bar = 100 μm). **(F)** H-scores depicting quantitative analysis of CD3 and Iba1 staining in brain tumor samples at endpoint from **(E)** as evaluated by blinded pathologist.
